# Mineralization mechanism and pollutant immobilization characteristics of vaterite induced by *Bacillus velezensis* and *Pseudomonas putida*

**DOI:** 10.3389/fmicb.2026.1966755

**Published:** 2026-10-13

**Authors:** Xiaofang Li, Wenjun Nie, Zhen Guo, Lin Tang, Fuguo Liu, Bin Lian

**Affiliations:** 1 Shandong Provincial University Laboratory for Protected Horticulture, Shandong Facility Horticulture Bioengineering Research Center, Weifang University of Science and Technology, Weifang, China; 2 College of Life Sciences, College of Marine Science and Engineering, Nanjing Normal University, Nanjing, China

**Keywords:** bacteria, biomineralization, copper tetracycline combined pollution, microbially induced carbonate precipitation, vaterite

## Abstract

Exploring strain-specific differences in bacterial vaterite biomineralization can supplement the basic theoretical research of biomineralization and reveal the biogeochemical linkages between microbially induced carbonate formation and in-situ pollutant sequestration in soil environments. In this study, *Bacillus velezensis* LB002 and *Pseudomonas putida* KT2440 were used as representative bacterial strains to compare strain-dependent vaterite formation and pollutant sequestration behavior. The biomineralization processes in the liquid medium were dynamically monitored, and the induced biominerals were characterized, with particular attention to their organic components. The differences in vaterite characteristics between both bacterial systems were evaluated, and their sequestration performance was assessed taking Cu^2+^ and tetracycline as target pollutants. Both strains induced vaterite formation under identical culture conditions, while *B. velezensis* exhibited a stronger biomineralization capacity than *P. putida*. In a 100 mL culture system, the Ca^2+^ mineralization percentage of *B. velezensis* reached 35.06%, and the dry weights of biomineral aggregates produced by *B. velezensis* and *P. putida* were 0.36 and 0.31 g, respectively. SEM observations revealed that the vaterite induced by *B. velezensis* had a loose and porous surface, whereas that induced by *P. putida* had a relatively smooth surface. For the high concentration of Cu^2+^ at 50 mg/L, the sequestration rate reached 84.93% for *B. velezensis*-induced vaterite, compared with 71.86% for *P. putida*-induced vaterite under the same conditions. Both biominerals demonstrated similar tetracycline sequestration efficiency, with values of approximately 60%. This study verifies that these two representative bacterial strains differ markedly in biogenic vaterite formation and mineral-mediated pollutant sequestration capacity, with interspecific variations in cell wall, EPS and CA activity accounting for these strain-dependent disparities. These findings help interpret why soil carbonate minerals show heterogeneous pollutant immobilization effects in natural edaphic environments, and offer experimental evidence for understanding microbe–carbonate–contaminant coupled biogeochemical cycling within soils and sediments.

## 1 Introduction

Microbially induced calcium carbonate precipitation (MICP) is a widespread biosedimentary process in both terrestrial and marine environments, where it contributes substantially to elemental biogeochemical cycling and the formation of marine sedimentary rocks (Dupraz et al., 2009; Diaz and Eberli, 2019; Wang et al., 2023). Under suitable environmental conditions, most naturally occurring microorganisms can mediate calcium carbonate synthesis (Hou et al., 2011; Li X. et al., 2023; Xiao and Lian, 2016; Whitaker et al., 2018). Among bacterially induced calcium carbonate biominerals, calcite, aragonite–calcite assemblages, and vaterite–calcite assemblages are the most frequently reported forms (Tourney and Ngwenya, 2009; Dhami et al., 2013; Lyu et al., 2021). The diversity of crystal polymorphs and morphologies produced during bacterial mineralization is controlled by several biological and geochemical processes. Bacterial cell walls may provide mineralization templates that promote calcium carbonate nucleation and crystal growth (Castanier et al., 2000; Li X. et al., 2023). Carbonic anhydrase (CA) secreted by bacteria accelerates the hydration of CO_2_ to HCO_3_^−^, which can subsequently be converted into CO_3_^2−^ under weakly alkaline conditions and incorporated into biomineralization (Castro-Alonso et al., 2019; Han et al., 2013; Zhang et al., 2023; Lyu et al., 2021). Extracellular polymeric substances (EPS) can regulate mineral formation because their negatively charged functional groups, including carboxyl, hydroxyl, and phosphate groups, can chelate Ca^2+^ and provide nucleation sites for biogenic calcium carbonate formation (Tourney and Ngwenya, 2014; Zhuang et al., 2018; Zhang et al., 2023). The type and abundance of organic matter generated by different bacteria during cultivation influence both the polymorph composition and structural stability of the resulting calcium carbonate biominerals (Zhu et al., 2021; Li X. et al., 2023). For instance, calcite and aragonite have relatively stable crystal structures and can be formed in the presence of comparatively simple organic components (Yu et al., 2018). Pure abiotic vaterite is thermodynamically metastable and readily transforms into calcite or aragonite in aqueous environments. In contrast, vaterite is thermodynamically metastable and hard to form under conventional inorganic conditions. Its nucleation generally relies on amino acids or small-molecule proteins (Hood et al., 2014; Zhang et al., 2020; Gao et al., 2023). In most abiotic calcium carbonate systems, metastable vaterite readily transforms into calcite or aragonite (Zhu et al., 2021; Li et al., 2019). However, biogenic vaterite from microbial systems can be stabilized by extracellular organic matrices to resist phase transition (Liu et al., 2021).

Previous studies have further confirmed the pollutant removal capacity of biogenic vaterite (BV). *Bacillus velezensis*-induced vaterite has demonstrated strong sequestration capacities for high concentrations of heavy metals, including Cd^2+^, Ni^2+^, and Cu^2+^, with maximum sequestration capacities (Qmax) of 172.41, 270.27, and 178.57 mg/g, respectively. These capacities are higher than those reported for conventional soil geochemical components such as biochar and graphene oxide (Park et al., 2016; Tan et al., 2015; Liu and Lian, 2019a,b). Biogenic vaterite can also sequester antibiotic contaminants such as doxorubicin hydrochloride, with a Qmax of 447.58 mg/g (Zhang et al., 2022), demonstrating its promising retention potential for organic pollutants. Benefiting from its large specific surface area, loose porous structure and excellent structural stability, BV is also applicable to drug loading (Li J. et al., 2023) and natural element cycling (Liu and Lian, 2019a,b; Jin et al., 2024). Nevertheless, the biomineralization mechanism, controlling factors, and practical applicability of BV remain insufficiently clarified. Most existing MICP and BV-related studies primarily focus on single-strain mineralization systems, while few investigations systematically compare interspecific differences in vaterite formation characteristics, mineral micromorphology, and pollutant immobilization efficiency. The intrinsic mechanisms underlying strain-specific biomineralization and pollutant sequestration remain poorly clarified, limiting our comprehensive understanding of bacterial-regulated vaterite biomineralization. In particular, the key factors dominating bacterial-induced BV formation, the regulatory mechanisms of BV morphology and particle size, and the pollutant sequestration potential of BV derived from different bacterial strains still need further exploration. Agricultural copper pollution originates from multiple sources, including chemical fertilizers, pesticides, and livestock feed additives. Copper-containing compound fertilizers and organic fertilizers have particularly pronounced effects on soil copper content (Xiao et al., 2022). Long-term and excessive application of organic fertilizers, such as livestock manure, not only intensifies copper accumulation in soils but also increases antibiotic concentrations, thereby threatening soil ecological quality and human health (Marti et al., 2013; Wang et al., 2013; Wang et al., 2021; Xu et al., 2023). Among various veterinary antibiotics, tetracyclines are frequently detected in manured agricultural soils and often co-occur with Cu, forming typical heavy metal-antibiotic combined contamination. Therefore, tetracycline was selected as the representative organic pollutant to evaluate the antibiotic sequestration performance of biogenic vaterite (Seiler and Berendonk, 2012; Cycon et al., 2019).

Published studies have demonstrated that Gram-positive *Bacillus* strains and Gram-negative *Pseudomonas* strains generally differ in EPS secretion and carbonic anhydrase production (Shirakawa et al., 2011; Liu et al., 2023), and our preliminary tests further confirmed such physiological disparities related to biomineralization. To evaluate the potential application of BV in agricultural production, *B. velezensis* LB002 (Gram-positive, G^+^) and *P. putida* KT2440 (Gram-negative, G^−^) were selected as experimental strains. Both strains are typical environmental functional bacteria widely distributed in natural soils, with similar ecological niches and strong environmental adaptability. Meanwhile, they exhibit distinct inherent differences in cell wall structure, as well as the composition and secretion characteristics of extracellular polymeric substances (EPS). This controllable comparative system enables an in-depth investigation into the effects of bacterial Gram characteristics and extracellular properties on the biosynthesis, micromorphology and mineralization efficiency of vaterite. Their capacity to induce BV synthesis and the temporal characteristics of mineralization were examined under identical culture conditions. The dry weight, micromorphology, and organic composition of BV produced by the two bacterial strains were compared. The roles of fermentation broth composition, physicochemical properties, and CA activity in bacterial biomineralization were analyzed. Finally, the removal efficiencies of BV induced by the two strains for organic and inorganic pollutants were assessed. The resulting evidence provides a basis for clarifying the strain-specific differences in BV formation and pollutant sequestration performance between the two tested bacterial strains and reveals the environmental geochemical potential of biogenic vaterite.

## 2 Materials and methods

### 2.1 Experimental strains and culture

*B. velezensis* LB002 (NCBI accession no. CP037417.1), previously isolated from soil (Li et al., 2025), and *P. putida* KT2440 (ATCC 33015), obtained as a model strain (Nelson et al., 2002), were used in this study. Both strains were preserved at the Key Laboratory of Microbiology and Functional Genes, Nanjing Normal University. Luria–Bertani (LB) medium was used for bacterial cultivation and mineralization induction. The medium was prepared with 1% (m/v) tryptone, 0.5% (m/v) yeast extract, and 1% (m/v) NaCl, and the initial pH was adjusted to 7.0–7.3. Bacterial cultures were incubated at 30 °C and 180 rpm in a shaking incubator. Each treatment was performed with three biological replicates.

### 2.2 Analysis of carbonate mineralization characteristics induced by two bacterial strains

Before mineralization experiments, both bacterial strains were activated in LB medium until the OD_600_ of the seed cultures reached approximately 1.2. The viable cell concentrations determined by plate counting were approximately 5 × 10^8^ CFU/mL for *B. velezensis* and 4 × 10^8^ CFU/mL for *P. putida*. For each mineralization treatment, 2 mL of the activated bacterial suspension was inoculated into 100 mL of LB medium supplemented with 0.8 g CaCl_2_. This calcium concentration was selected based on the previous vaterite induction results obtained by our research group (Liu et al., 2021). An abiotic control was established using LB medium containing 0.8 g CaCl_2_ without any bacterial inoculation. No visible precipitate was found in the abiotic control at all sampling time points during the entire 14-day incubation, ruling out spontaneous mineral precipitation caused by simple chemical mixing of LB medium and CaCl₂. All cultures and controls were incubated at 30 °C and 180 rpm for 14 d. Samples were collected after 1, 3, 5, 7, 10, and 14 d of incubation. The pH of each fermentation broth was measured using a pH meter (PHSJ-4F, Leici, China). The collected samples were centrifuged at 8000 rpm, and the supernatants were retained for Ca^2+^ analysis. The Ca^2+^ concentrations in the experimental and control groups were determined using atomic absorption spectroscopy (AA-6300C, Shimadzu, Japan). The Ca^2+^ mineralization percentage was calculated using the following equation (Li X. et al., 2023).


$$\begin{array}{l} \text{Percent of}\;{\mathrm{Ca}}^{2+}\text{mineralization}\;(\%)=\\ \frac{C_{{\mathrm{Ca}}^{2+}\text{in}\;\;\text{control}\;\;\text{group}}-C_{{\mathrm{Ca}}^{2+}\text{in}\;\;\text{experimental}\;\;\text{group}}}{C_{{\mathrm{Ca}}^{2+}\text{in}\;\;\text{control}\;\;\text{group}}}\times 100 \end{array}$$

The mixture was centrifuged at 8000 rpm for 5 min to separate the supernatant and precipitate. The precipitate was washed three times with double-distilled water, then dried in an oven at 55 °C for 12 h until constant weight was achieved. The dry mass of each precipitate was measured using an analytical balance. The mineral phases of the dried products were identified using X-ray diffraction (XRD; BTX-526, Olympus, USA) with a Co target over a scanning range of 5–55°.

### 2.3 Comparative analysis of carbonate mineralization ability and pollutant immobilization performance induced by two bacterial strains

#### 2.3.1 Comparison of organic composition and content of two biogenic vaterite

Preliminary tests using samples collected at different incubation stages confirmed that the calcium carbonate phase stabilized on day 7. At this stage, only slight changes in dry weight were observed, and the Ca^2+^ mineralization percentage reached an approximately steady state. Accordingly, the day-7 mineral products were selected for subsequent comparative analysis. After 7 d of incubation, the precipitates were collected, washed, and dried to a constant weight. The organic elemental compositions of the two biomineral aggregates were determined using an elemental analyzer (vario EL cube, Elementar, Germany). Fourier transform infrared spectroscopy (FTIR; Nicolet 8,700, Thermo Nicolet, USA) and thermogravimetry–derivative thermogravimetry (TG-DTG; STA7300, Perkin-Elmer, USA) were used to identify the organic matter composition and quantify its content in the biominerals (Liu and Lian, 2019b; Li X. et al., 2023).

#### 2.3.2 Comparison of morphology of two biogenic vaterite samples

Following centrifugation and lyophilization, the biomineral powders were characterized using scanning electron microscopy coupled with energy-dispersive spectroscopy (SEM–EDS; Supra 55, Zeiss, Germany). Transmission electron microscopy (TEM; HT7700, Hitachi, Japan) was used to examine the microstructure and crystal morphology of the induced biominerals. For TEM preparation, 1.5 mL of bacterial fermentation broth was centrifuged, and the supernatant was discarded. The collected bacterial cells were fixed in 2.5% glutaraldehyde fixative for electron microscopy, embedded in epoxy resin, and sectioned into ultrathin slices using a microtome (EMUC7, Leica, Germany). The prepared sections were subsequently used for TEM observations and analyses.

### 2.4 Analysis of biochemical characteristics of bacterial culture solution during bacteria-induced mineralization

After 7 d of cultivation, the fermentation broths from the two bacterial strains were centrifuged, and the collected supernatants were subjected to biochemical analysis. Polysaccharide content was quantified using the phenol–sulfuric acid method, and protein content was measured using a Bradford Protein Assay Kit (Shanghai Sangon Biotech, China) (Li X. et al., 2023). The ability of the fermentation supernatants to induce calcium carbonate formation was examined using a biomimetic mineralization system established according to previous methods (Lian et al., 2006; Liu et al., 2021). Excess NH_4_HCO_3_ was placed at the bottom of the mineralization device, and 20 mL of a mixed solution containing 0.2 mol/L CaCl_2_ and NaHCO_3_ was introduced into the upper layer of each group. Parallel mineralization systems were supplemented with 5 mL of fermentation supernatant from each bacterial strain or 5 mL of double-distilled water. The sealed devices were maintained at room temperature for 14 d. The minerals formed during incubation were collected and analyzed using XRD for crystal phase identification (Lian et al., 2006; Liu et al., 2021). The carbonic anhydrase (CA) activity in the bacterial supernatants was also determined to further compare the mineralization-inducing capacity of the two strains. The working solution for CA assay was prepared by mixing 1 mL of 0.0036 g/mL p-nitrophenyl acetate with 10 mL of 0.00156 g/mL methylmalonic acid. Then, 500 μL of centrifuged supernatant was mixed with 500 μL of the above-mentioned working solution, and the reaction mixture was incubated in a water bath at 35 °C for 30 min. The reaction was terminated in an ice-water bath, followed by centrifugation at 4 °C and 12,000 rpm for 3 min. The absorbance was measured at 400 nm using a multifunctional microplate reader (Synergy2, BioTek, USA). Parallel blank controls using ultrapure water were included. The enzyme activity was calculated according to the standard curve Y = 9.35X + 0.03, where Y is the optical density at 400 nm and X is the corresponding p-nitrophenol concentration (Li and Lian, 2023). After centrifugation, the biomineral precipitates were dried to a constant weight, and their carbonate content was determined by acid–base titration (Li X. et al., 2023).

### 2.5 Pollutant sequestration experiment of biogenic vaterite toward simulated single and composite pollutants

Biominerals harvested after 7 d of incubation were used to evaluate the sequestration performance of biogenic vaterite for simulated inorganic and organic pollutants, represented by Cu^2+^ and tetracycline (TC), respectively. Single-pollutant solutions were prepared at Cu^2+^ concentrations of 10, 20, 30, 40, and 50 mg/L and TC concentrations of 5, 10, 15, 20, and 25 mg/L. Combined pollutant solutions containing both Cu^2+^ and TC were prepared using the same concentration gradients. The initial pH of all single and combined pollutant solutions was adjusted uniformly to 5.0 before adsorption experiments. For each treatment, 20 mL of the pollutant solution was placed in a 50 mL centrifuge tube, and 0.05 g of biogenic vaterite induced by either bacterial strain was added. The mixtures were shaken at 25 °C and 120 rpm for 12 h, followed by centrifugation at 8000 rpm to separate the supernatant and precipitate. The residual Cu^2+^ concentration in the supernatant was measured using atomic absorption spectroscopy (AAS), and the residual TC content was calculated from the absorbance at 415 nm using a spectrophotometer (Rathod et al., 2015). The pore structural properties of vaterite induced by the two bacteria were preliminarily examined through N_2_ adsorption–desorption isotherm analysis using an automatic gas adsorption analyzer after degassing at 80 °C for 12 h. The specific surface area was calculated using the BET model, the core theoretical model for specific surface area determination, whereas the pore size distribution was obtained using the BJH model, a classical model for 2–50 nm mesoporous pore size analysis (Wang et al., 2014; Sheng et al., 2009).

### 2.6 Statistical analysis

All experiments in this study were performed with three independent biological replicates. Prior to statistical testing, Shapiro–Wilk test and Levene’s test were separately conducted to assess the normality and homogeneity of variance (variance structure) of all datasets. One-way analysis of variance (ANOVA) was used to test whether the overall differences between groups were statistically significant, followed by Tukey’s HSD multiple comparison test to further determine which specific groups had significant differences. All statistical analyses were completed using IBM SPSS Statistics 20.

## 3 Results

### 3.1 Synthesis process and capacity analysis of vaterite induced by two bacterial strains

The fermentation media of the two bacterial strains were dynamically monitored to characterize vaterite formation. The pH of both fermentation broths increased gradually from day 1 to day 3. During days 3–5, massive vaterite precipitation in the *B. velezensis* system consumed OH^−^, leading to a slight pH decrease at day 5, whereas the *P. putida* group maintained continuous alkalization. By day 7, the pH stabilized at a weakly alkaline level, reaching approximately 8.10 for *B. velezensis* and approximately 8.30 for *P. putida* (Figure 1a). Consistent with this pH evolution, the Ca^2+^ mineralization percentages in both bacterial systems approached equilibrium by day 7 (Figure 1b). The measured Ca^2+^ balance indicated that only a small fraction of the consumed Ca^2+^ was directly assimilated by bacterial cells, whereas most of the consumed Ca^2+^ was immobilized in newly formed biominerals. The precipitate dry weight of *P. putida* reached a steady state on day 3, indicating rapid saturation of mineral formation, which is consistent with the early detection of vaterite in the XRD analysis (Figure 2b). In contrast, precipitate dry weight in the *B. velezensis* system increased continuously during the first 7 d and then remained nearly constant (Figure 1c). Based on the results shown in Figure 1b, the Ca^2+^ mineralization percentage reached approximately 35.06% for *B. velezensis* on day 7, compared with 27.50% for *P. putida*. The corresponding dry weights of the biomineral aggregates were 0.36 and 0.31 g, respectively (Figure 1c). Therefore, the temporal variation in precipitate dry weight was consistent with the change in the Ca^2+^ mineralization percentage (Figures 1b,c).

**Figure 1:**
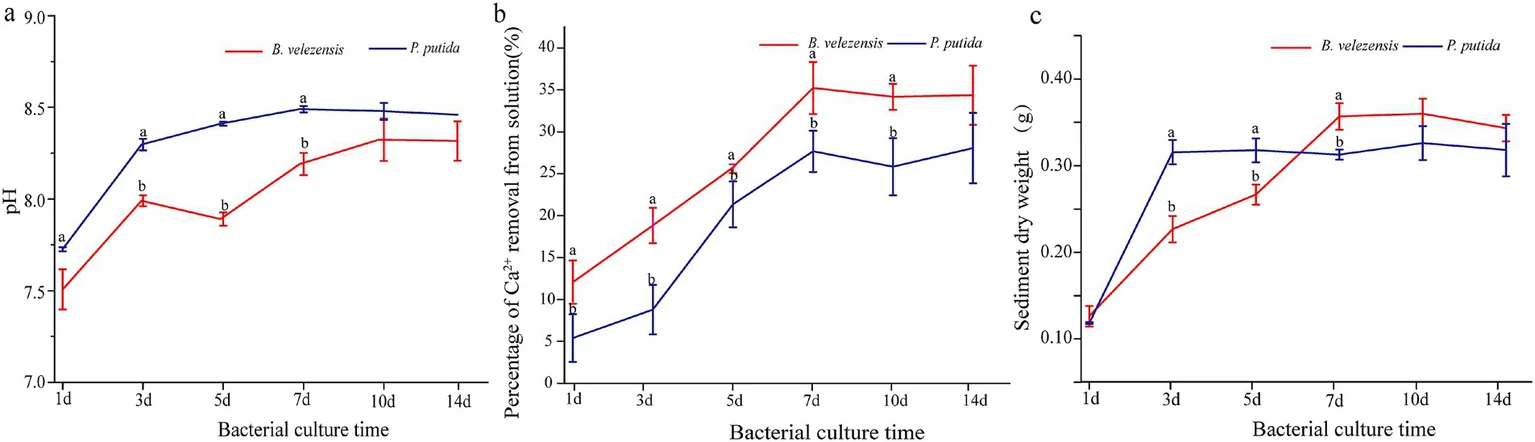
Dynamic changes in key mineralization parameters induced by the two bacterial strains. **(a)** Variation in pH during the bacterial-induced mineralization process; **(b)** comparison of Ca^2+^ mineralization rates induced by the two bacterial strains; **(c)** comparison of the dry weights of biominerals formed by the two bacterial strains. Different lowercase letters a and b above data points indicate significant differences between the two strains at the same sampling time. No labels are displayed for time points without significant differences (one-way ANOVA, *p* < 0.05).

**Figure 2:**
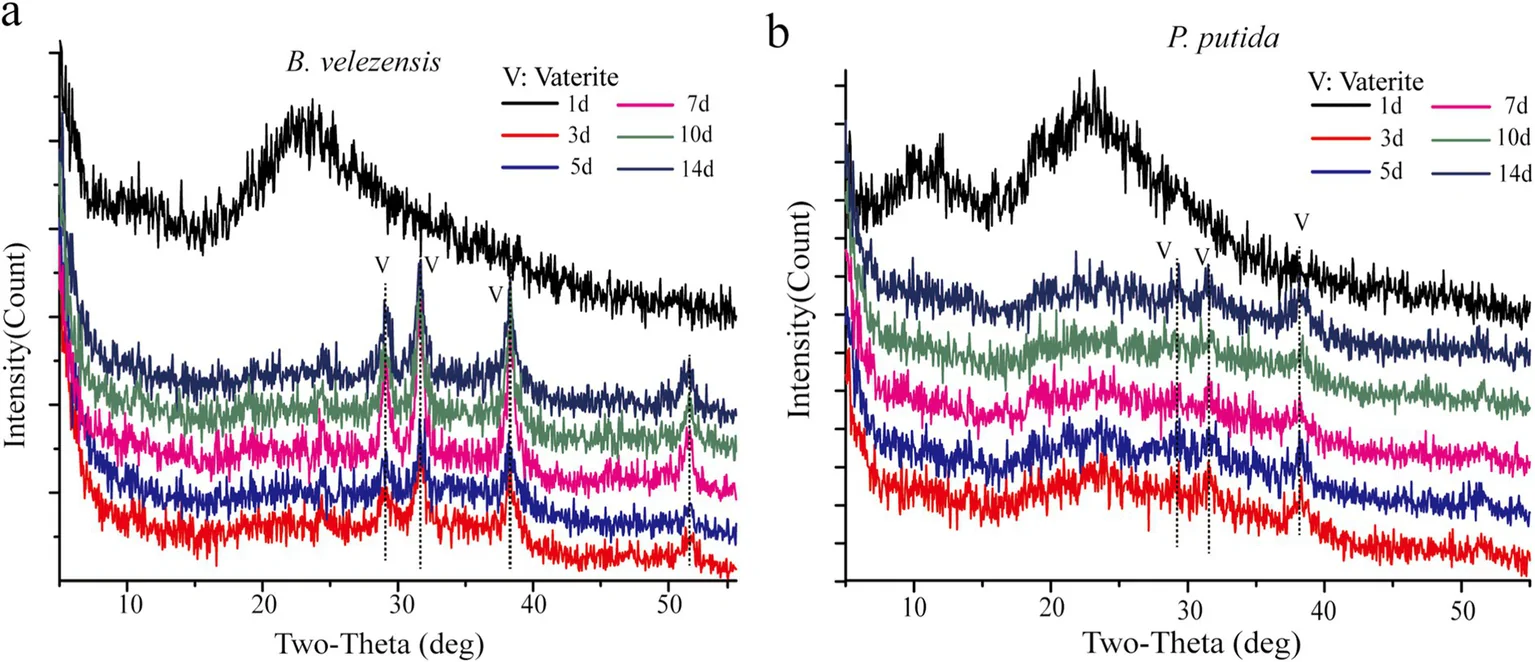
XRD patterns of mineral products induced by different bacterial strains during 1–14 d of incubation. **(a)** Identification of mineral products induced by *B. velezensis*; **(b)** identification of mineral products induced by *P. putida*.V, vaterite.

XRD analysis was conducted to identify the mineral phases of the biominerals induced by the two bacterial strains. No biomineral products were detected in either bacterial system on day 1(Figure 2a). From day 3 onward, both *B. velezensis* and *P. putida* induced vaterite formation, although the vaterite diffraction peaks in the *P. putida* were relatively weak. With increasing incubation time, the crystal phase of the induced carbonate minerals remained unchanged, and vaterite was maintained as the dominant phase throughout the cultivation period (Figures 2a,b).

### 3.2 Organic matter content and mineral characterization of vaterite induced by two bacterial strains

In this study, the N, C, and H contents of the biomineral samples were used to evaluate the organic fraction within the mineral aggregates (Figure 3a). The higher N and C contents suggest a higher proportion of unmineralized bacterial cells or organic constituents retained in the organic–inorganic mineral composites. This composition was accompanied by a lower relative calcium carbonate fraction and reflected weaker Ca^2+^ mineralization capacity (Figure 1b). Elemental analysis of the day-7 biomineral aggregates indicated that the *B. velezensis*-induced vaterite contained 4.02% N, 20.89% C, and 3.27% H, whereas the *P. putida*-induced vaterite contained 6.49% N, 27.74% C, and 4.78% H. The higher elemental content measured in the *P. putida*-derived product indicated greater organic matter retention in this biomineral (Figure 3a). In addition, FTIR characterization supported the incorporation of organic components into both biogenic vaterites. Characteristic bands assigned to -OH, -CH, and -SH were observed at 3412, 2968, and 579 cm^−1^, respectively (Figure 3b), confirming the presence of abundant polysaccharides and proteins. Meanwhile, typical vaterite bands were detected at 1083, 876, and 747 cm^−1^ (Liu and Lian, 2019b). The organic matter content of the biomineral aggregates collected after 7 d of incubation was quantified using TG-DTG analysis(Figures 3c,d). Because large numbers of bacterial cells were present in the precipitates, the detected organic fraction included both organic substances incorporated into the mineral matrix and residual free bacterial cells that had not been fully mineralized. The corresponding results are shown in Figure 3c,d. The thermal mass-loss profiles of the two biominerals were divided into three stages. The first stage, occurring near 200 °C, corresponded to the evaporation of bound water. The second stage, extending from 201 to 618 °C, was mainly associated with the combustion of organic matter. The third stage appeared at approximately 600 °C and represented biomineral decomposition, during which calcium carbonate began to decompose into CaO and CO_2_. According to the mass losses recorded across these stages, the organic matter contents of the biominerals induced by *B. velezensis* and *P. putida* were calculated as 27.50% and 37.59%, respectively. The higher organic matter content in the *P. putida*-induced biomineral aggregates (Figures 3a,d) suggests a weaker mineralization capacity and greater retention of residual organic components, including bacterial cells, in the precipitates. In contrast, *B. velezensis* exhibited a stronger mineralization ability and produced precipitates with a higher calcium carbonate proportion. This pattern was consistent with the elemental analysis results (Figure 3a).

**Figure 3:**
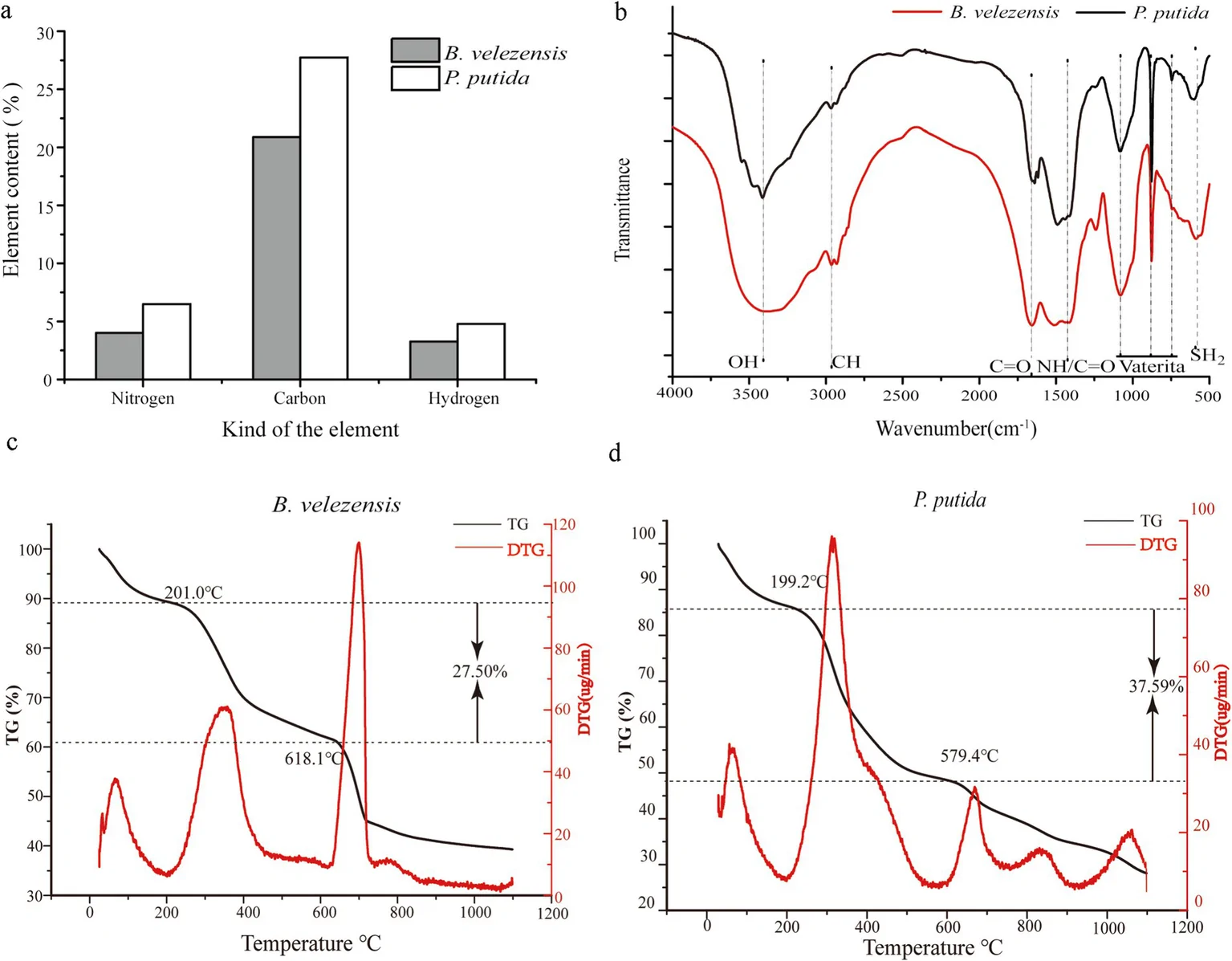
Characterization of the types and contents of organic matter in biogenic vaterite. **(a)** Major elemental compositions of the two biomineral samples; **(b)** FTIR spectra of the two biomineral samples; **(c)** thermogravimetric analysis of biominerals induced by *B. velezensis*; **(d)** thermogravimetric analysis of biominerals induced by *P. putida*.

### 3.3 Morphological comparison of vaterite induced by two bacterial strains

After vaterite formation was confirmed by XRD, SEM analysis was conducted to compare the morphology of the mineral products induced by the two bacterial strains. The *B. velezensis*-induced products were composed of micro- to nanoscale vaterite particles with smaller particle sizes and a loose surface texture (Figures 4a,b). The participation of extracellular organic matter in the mineralization process was reflected by the incorporation of bacterial cells and spores into the mineral phase, resulting in compact bacteria–mineral complexes (Figures 4a,c). In contrast, the products induced by *P. putida* were dominated by relatively larger particles at the micron scale, and the corresponding biomineral complexes contained a higher proportion of organic matter (Figures 4d,e). EDS analysis indicated that the *P. putida*-induced vaterite was characterized by higher carbon content and lower calcium content than the *B. velezensis*-induced vaterite (Figure 4f). This revealed relatively lower degree of carbonate mineralization and confirmed the stronger mineralization-inducing capacity of *B. velezensis*.

**Figure 4:**
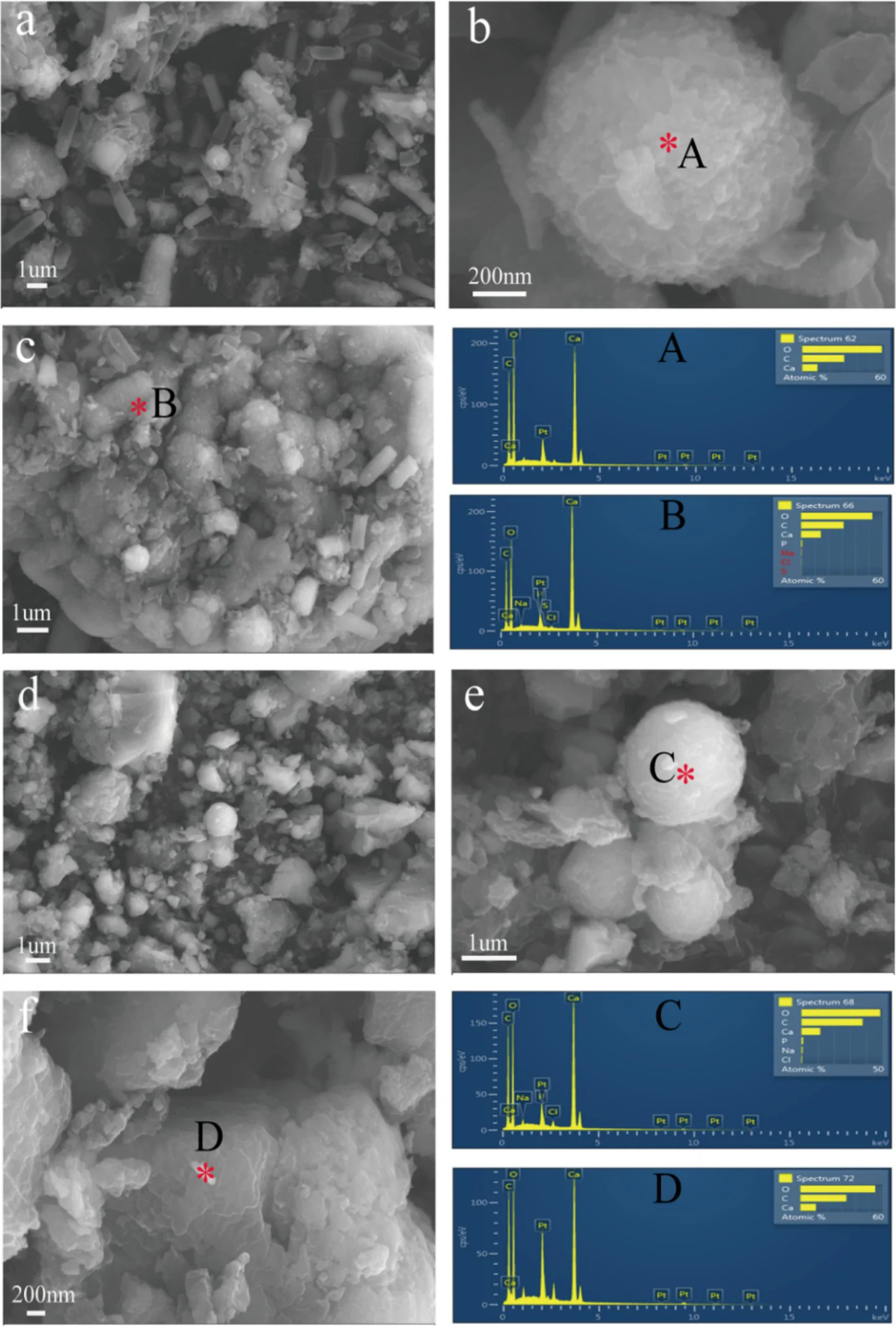
SEM-EDS characterization of biogenic vaterite induced by the two bacterial strains. **(a–c)** SEM morphologies of biominerals induced by *B. velezensis*. Spectrum A corresponds to the region marked with an asterisk in panel **(b)**; Spectrum B corresponds to the region marked with an asterisk in panel **(c)**. **(d–f)** SEM morphologies of biominerals induced by *P. putida*. Spectrum C corresponds to the region marked with an asterisk in panel **(e)**; Spectrum D corresponds to the region marked with an asterisk in panel **(f)**.

Surface-associated mineralization of the two bacterial strains was examined using TEM. Supplementary Figure S1a shows the cellular ultrastructure of *B. velezensis* and *P. putida* at day 7, revealing inherent differences in cell surface and cell-wall features between the Gram-positive and Gram-negative strains. *B. velezensis* retained a rod-shaped morphology, and numerous acicular mineral particles were distributed around the bacterial cells (Supplementary Figure S1a,b). The *P. putida* cells appeared spherical, with biomineral material attached to their surface (Supplementary Figure S1c,d). However, the selected area electron diffraction (SAED) analysis of the mineralized surfaces of both bacterial strains did not reveal characteristic diffraction patterns corresponding to vaterite.

### 3.4 Analysis of biochemical characteristics of culture solution related to bacterial mineralization

The carbonic anhydrase (CA) activity differed markedly between the two bacterial fermentation broths. The CA activity measured in the *B. velezensis* broth was approximately five times higher than that in the *P. putida* broth (Figure 5a). Consistent with this difference, titration analysis demonstrated that the carbonate proportions in the biominerals induced by *B. velezensis* and *P. putida* were 38.93% and 24.26%, respectively (Figure 5b). The biochemical compositions of the 7-day fermentation broths were analyzed. *B. velezensis* secreted a significantly higher polysaccharide content than *P. putida*, with values of 0.53 and 0.33 g/L, respectively (Figure 5c). Conversely, the protein content in *P. putida* reached 0.024 g/L, which was higher than that of 0.014 g/L in *B. velezensis* (Figure 5d). Subsequently, biomimetic mineralization experiments were performed using fermentation supernatants from both bacterial strains, along with dextran and bovine serum albumin. The fermentation broths of both *B. velezensis* and *P. putida* partially converted calcite to vaterite, whereas ultrapure water did not induce this phase transformation (Supplementary Figure S2). These results suggest that CA promotes the generation of carbonate ions, thereby facilitating their subsequent binding with calcium ions to form calcium carbonate. Protein components may contribute to vaterite biosynthesis and stabilization during mineralization.

**Figure 5:**
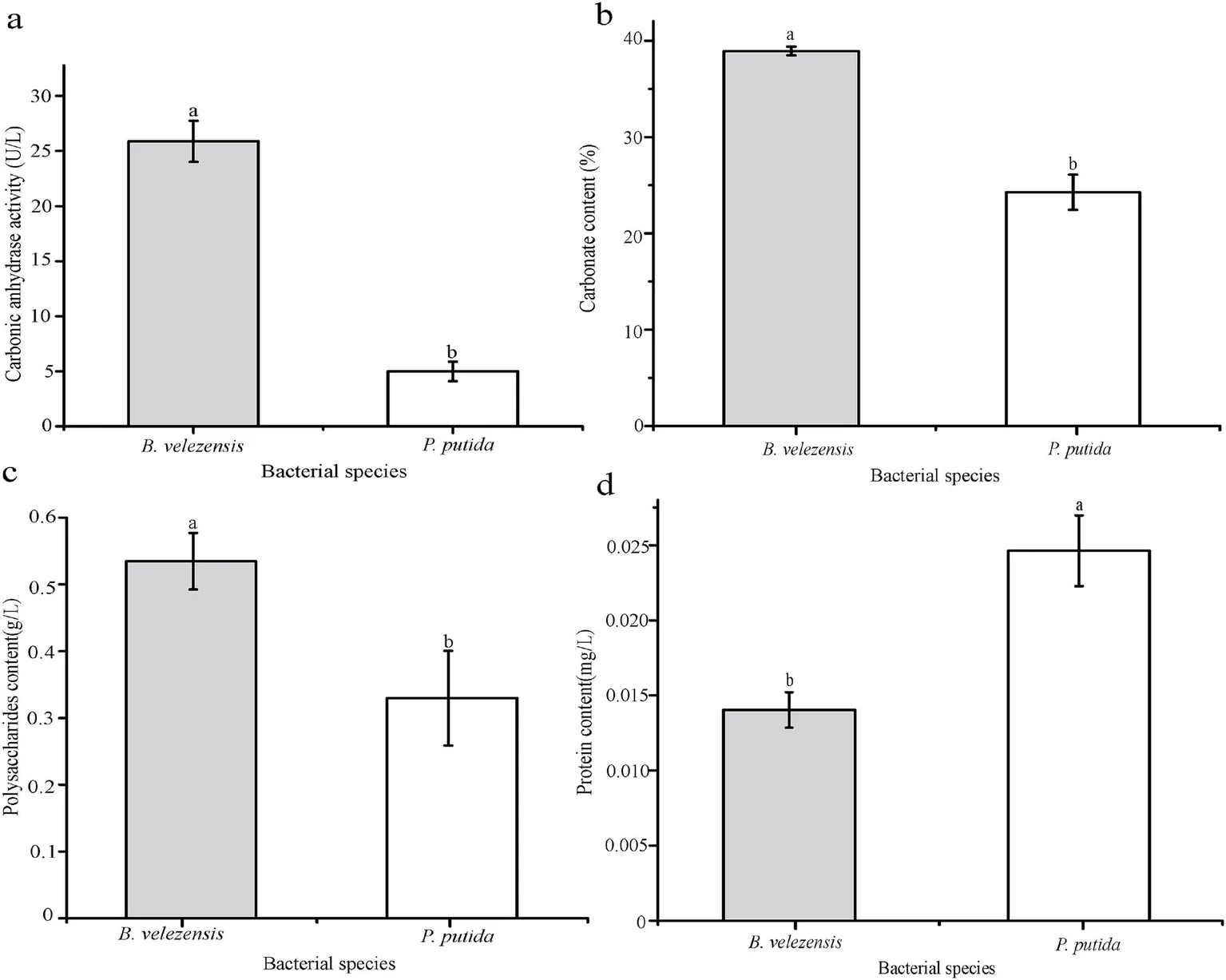
Mechanistic analysis of differences in carbonate mineralization induced by the two bacterial strains. **(a)** Determination of carbonic anhydrase activity; **(b)** carbonate content of biominerals; **(c)** polysaccharide content in the fermentation broth; **(d)** protein content in the fermentation broth. Different lowercase letters above the bars indicate significant differences between the two strains (*p* < 0.05).

### 3.5 Pollutant sequestration characteristics of biogenic vaterite formed by two bacterial strains

The environmental application potential of the two biogenic vaterites was assessed by comparing their sequestration efficiency for inorganic and organic pollutants. In both single- and combined-pollutant systems, *B. velezensis*-induced vaterite showed a stronger capacity for Cu^2+^ removal than *P. putida*-induced vaterite. At a high Cu^2+^ concentration of 50 mg/L, the removal efficiencies of the *B. velezensis*-induced vaterite reached 84.37% and 84.93%, respectively (Figures 6a,b), whereas those of the *P. putida*-induced vaterite were 69.16 %. and 71.86% under the same conditions. The limited difference between the single and combined systems indicates that the introduction of TC exerted only a minor effect on Cu^2+^ adsorption by vaterite. At lower Cu^2+^ concentrations (10–20 mg/L), the *B. velezensis*-induced vaterite maintained higher removal efficiencies than the *P. putida*-induced vaterite. This stronger heavy metal sequestration efficiency was associated with its larger specific surface area and pore structure, with a specific surface area of 13.19 m^2^/g and an average pore size of 4.88 nm, compared with 2.56 m^2^/g and 4.13 nm for the *P. putida*-induced vaterite, respectively (Supplementary Table S1). In the single TC system, the *P. putida*-induced vaterite presented a higher TC sequestration efficiency at the low TC concentration of 5 mg/L, reaching 64.94% compared with 49.08% for the *B. velezensis*-induced vaterite. As the TC concentration increased, the removal efficiencies of both biominerals increased and gradually approached approximately 70% (Figure 6c). In the combined-pollutant system, Cu^2+^ addition reduced TC removal by both types of biogenic vaterite (Figure 6d). This reduction was mainly attributed to the preferential occupation of binding sites by Cu^2+^, whereas TC, with its relatively larger molecular size, exhibited weaker competitive access to these sites. Overall, *B. velezensis*-induced vaterite exhibited more favorable sequestration efficiency, particularly for Cu^2+^ immobilization.

**Figure 6:**
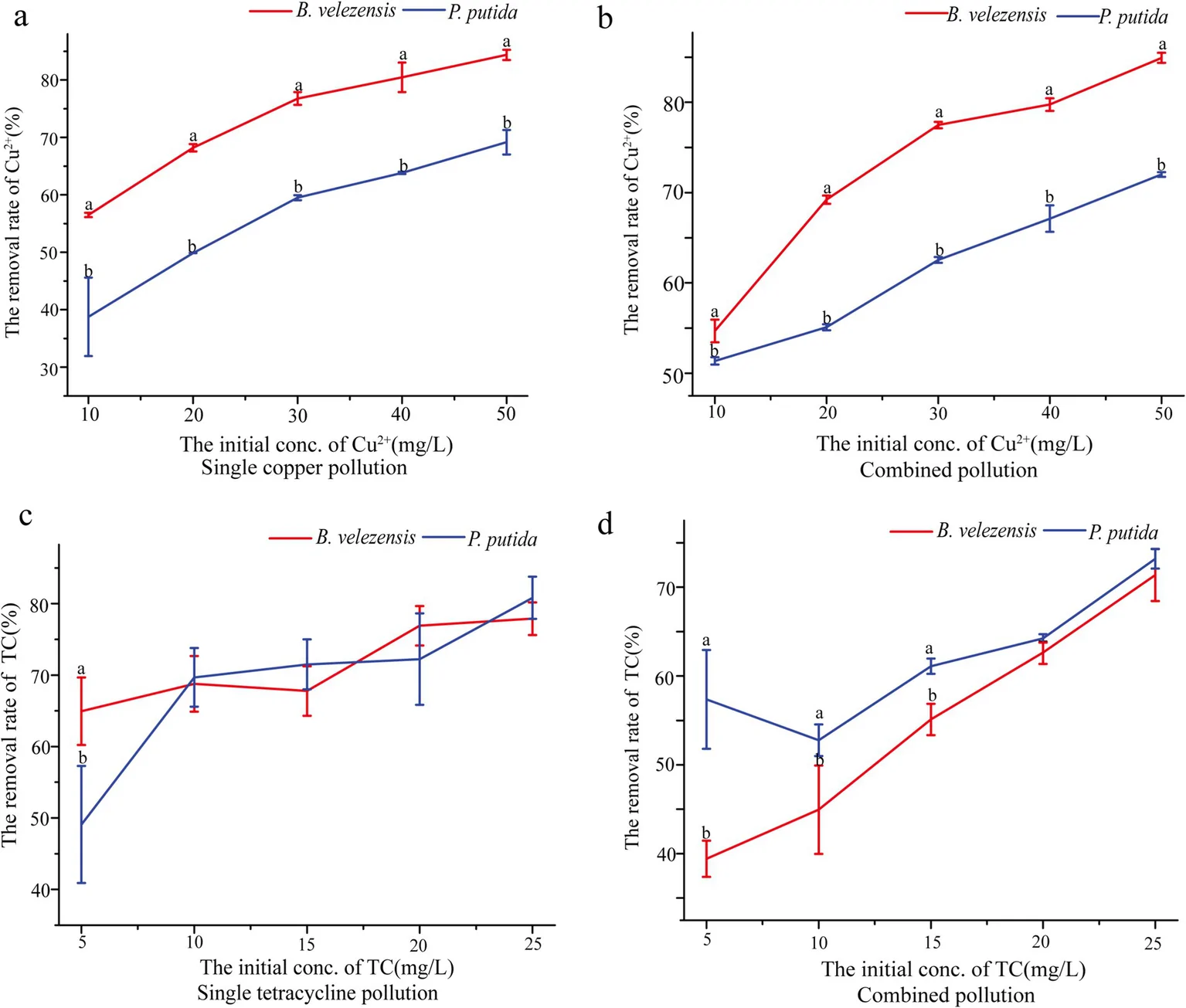
Pollutant sequestration characteristics of biogenic vaterite produced by the two bacterial strains. **(a)** Cu^2+^ sequestration efficiency under single-pollutant system; **(b)** tetracycline (TC) sequestration efficiency under single-pollutant system; **(c)** Cu^2+^ sequestration efficiency under combined pollution system; **(d)** TC sequestration efficiency under combined pollution system. Different lowercase letters above bars indicate significant differences at *p* < 0.05. No labels are displayed for time points without significant differences.

## 4 Discussion

### 4.1 Key controlling factors for bacterially induced Vaterite synthesis

*B. velezensis* is a Gram-positive (G^+^) bacterium with surface flagella and the ability to form endospores. It commonly colonizes soil and plant surfaces and is widely used as a probiotic strain in agricultural production(Geng et al., 2026). *P. putida* is a Gram-negative (G^−^), ovoid, lophotrichous bacterium that is broadly distributed in natural environments. Although both strains differ markedly in cell type and physiological characteristics, they are capable of inducing biogenic vaterite formation in Ca^2+^-supplemented culture systems. During mineralization, the pH increased from day 1 to day 3 of incubation (Figure 1a). This early alkaline shift may be partially associated with CO₂ conversion to HCO₃^−^, which could be catalyzed by carbonic anhydrase (CA) secreted by bacteria(Liu et al., 2021). The most rapid increase in precipitate dry weight also occurred during this period, followed by stabilization on day 7 (Figure 1c). However, the mineralization efficiency differed substantially between these two strains. The Ca^2+^ mineralization ratio reached 35.06% in the *B. velezensis* system, whereas only 27.50% was achieved in the *P. putida* (Figure 1b). This difference implies that *B. velezensis* provides a more favorable biological environment for biogenic vaterite formation. The stronger mineralization performance of *B. velezensis* was closely associated with differences in bacterial cell wall structure and extracellular polymeric substances (EPS). Cell wall characteristics and EPS profiles show correlations with the crystal phase and micromorphology of biogenic calcium carbonate. Existing studies proposed that proteins may serve as potential nucleation sites for vaterite crystallization (Addadi et al., 2006; Hood et al., 2014; Zhang et al., 2021; Gao et al., 2023), which is consistent with the correlative trends observed in our work. Although both strains induced vaterite under identical culture conditions, their mineral products exhibited evident morphological differences. *B. velezensis*-induced vaterite exhibited a loose, porous, and layered surface structure. The polysaccharides secreted by *B. velezensis* appeared to promote the binding of vaterite particles to bacterial cells, resulting in compact bacteria–mineral complexes (Figure 4c). In contrast, the *P. putida*-induced vaterite presented a smoother surface and lacked a layered microstructure. The lower polysaccharide content in the *P. putida* culture system weakened cell–mineral adhesion and restricted the formation of large bacteria–mineral aggregates (Figure 4d). These morphological differences imply a potential link between vaterite microstructure and the composition, category and relative abundance of EPS in the culture system.

The differences in carbonic anhydrase activity and polysaccharide production between the two strains mainly originated from their inherent genetic characteristics. Mineral samples were gently rinsed prior to characterization to remove suspended free cells and intracellular contents, ensuring that the detected organic matter such as proteins was mainly cell-surface associated substances rather than components released from disrupted biomass. Apart from EPS composition, pH shifts and CA activity exhibit measurable associations with the progression of calcium carbonate formation (Figures 1a,5a). During vaterite formation, proteins act mainly as nucleation sites for crystal initiation, whereas polysaccharides mediate adhesion between bacterial cells and mineral particles (Li X. et al., 2023), thereby promoting the development of bacteria–mineral aggregates (Supplementary Figure S2), while polysaccharides primarily promoted mineral-particle adhesion and aggregate enlargement (Figure 4b). This pattern is consistent with the findings of Zhang et al. (2020) and supports the involvement of EPS in fermentation broth-mediated biomineralization. Given the high compositional complexity of bacterial fermentation broth, components other than polysaccharides and proteins, such as organic acids, may participate in regulating the formation of different calcium carbonate polymorphs. Therefore, further studies are needed to clarify how the content and composition of polysaccharides and proteins modulate calcium carbonate morphology and bacteria–mineral complex formation.

Taken together, variations in vaterite biosynthesis between strains are correlated with cell wall features, extracellular metabolites and culture conditions (Figure 7). Both strains were able to induce biogenic vaterite under suitable culture conditions, and the mineralization process was closely related to pH variation and CA activity. Nevertheless, *B. velezensis* presented a stronger mineralization tendency and higher mineralization capacity. Extracellular metabolites (especially proteins and polysaccharides) exhibit strong correlations with extracellular biomineralization behavior. Proteins promoted vaterite nucleation and formation, whereas polysaccharides strengthened biomineral particle adhesion and supported the development of larger bacteria–mineral complexes. Consequently, although both strains produced biogenic vaterite, the resulting minerals differed markedly in terms of surface microstructure and pollutant immobilization performance.

**Figure 7:**
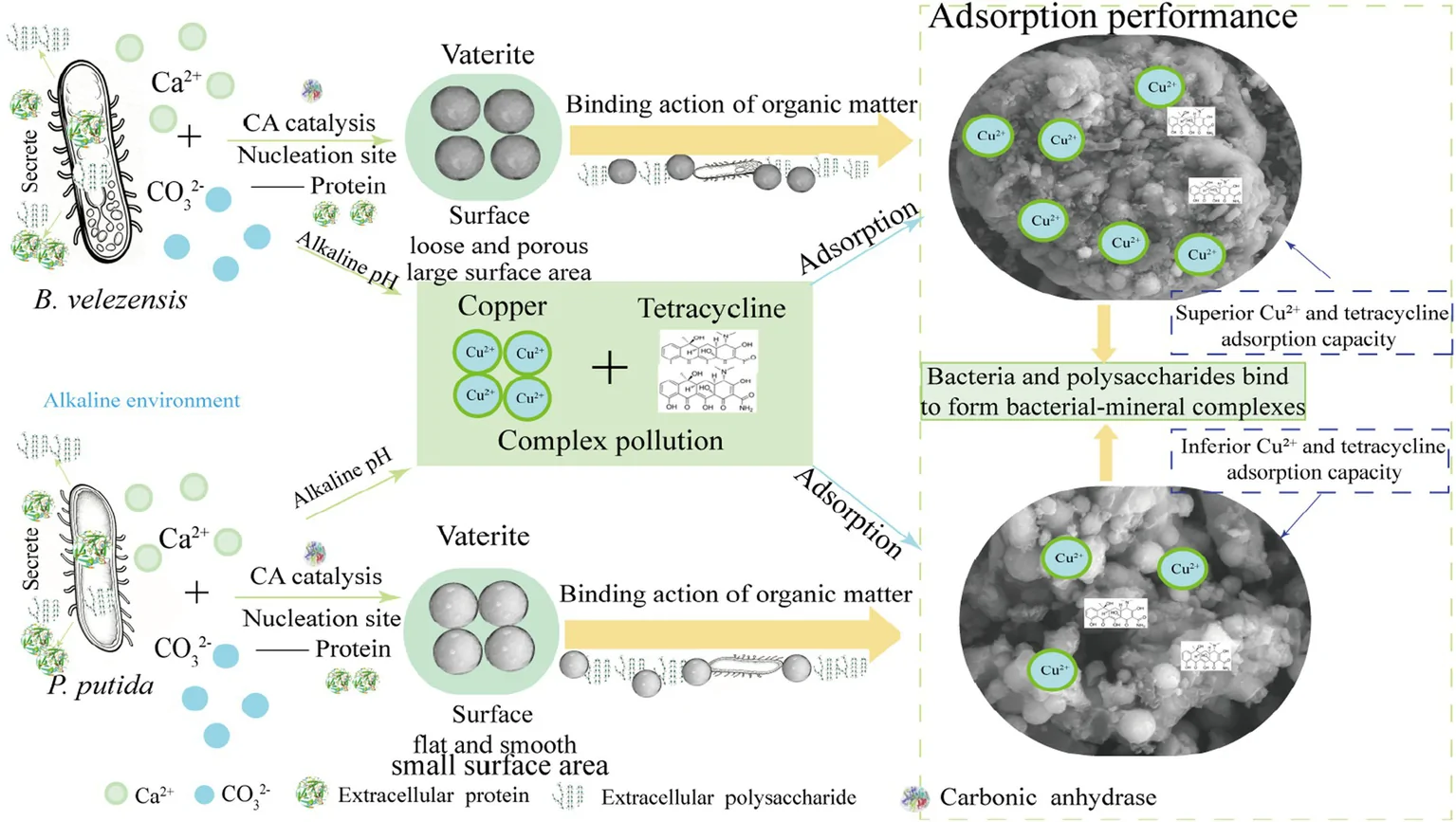
Proposed schematic model of calcium carbonate mineralization and combined pollutant immobilization induced by the two bacterial strains.

### 4.2 Synthesis mode of biogenic Vaterite

Compared with chemically synthesized vaterite, bacterially induced biogenic vaterite generally exhibits more complex morphology and higher structural stability, making it less prone to transformation into aragonite or calcite (Liu et al., 2021). This difference is mainly associated with the involvement of abundant organic matter, especially protein components, which can function as nucleation sites during vaterite formation. Both *B. velezensis* and *P. putida* secreted substantial EPS, including polysaccharides and proteins(Figures 5c,d). However, the higher CA activity of *B. velezensis* promoted greater Ca^2+^ precipitation as calcium carbonate in the culture system (Figure 5a). In addition, the two strains exhibit distinct cell wall architectures: *Bacillus velezensis* possesses a thick peptidoglycan layer typical of Gram-positive bacteria, whereas *Pseudomonas putida* has the thin cell wall structure characteristic of Gram-negative species. Previous studies speculated that peptidoglycan, as well as carboxyl and phosphate functional groups on teichoic acids, may interact with free Ca^2+^ and serve as potential mineralization templates (Zhang et al., 2021; Li X. et al., 2023). Nevertheless, cell wall structural divergence constitutes merely one of many physiological and structural disparities between these two phylogenetically distant strains. In fact, the divergent mineralization phenotypes detected in our system correlate with the combined effects of strain-specific carbonic anhydrase activity, EPS secretion patterns and inherent metabolic differences (Figure 7). Thermogravimetry-derivative thermogravimetry (TG-DTG) results reveal that precipitates produced by *Pseudomonas putida* exhibit stronger total organic signals(Figures 3c,d). Such organic matter likely originates from three major sources: residual intact bacterial cells or unmineralized organic substances attached to the surface of mineral aggregates, as well as partial organic components embedded within the vaterite crystal framework (Zapata-Martínez et al., 2025). Macroscopic thermal analysis and elemental detection alone cannot distinguish lattice-incorporated organic matter from bacterial debris adhering to mineral surfaces. Quantitative carbonate titration indicates that the carbonate content of vaterite biomineralized by *Pseudomonas putida* is approximately 14.7% lower than that of products generated by *Bacillus velezensis* (Figures 2a, 5b). This observation demonstrates a larger proportion of biological residues within the mineral aggregates of the former strain. Combined with the calculated calcium mineralization efficiencies (35.06% for *B. velezensis* and 27.50% for *P. putida*), these data prove that *Bacillus velezensis* possesses a higher calcium mineralization rate and thus superior biomineralization capacity (Figure 1b). For Cu^2+^ pollution, *B. velezensis*-induced biogenic vaterite had micro- to nanoscale particles and a large specific surface area, which facilitates immobilization of heavy metal ions (Liu et al., 2019). In contrast, the *P. putida*-induced vaterite had a smaller specific surface area and weaker Cu^2+^ adsorption performance (Figures 6a,b). For TC pollution, the removal efficiencies of biogenic vaterite induced by *B. velezensis* and *P. putida* differed only slightly, and both materials effectively reduced the TC concentration (Figures 6c,d). The large surface pore size and specific surface area of bacterially induced biogenic vaterite determine its natural capacity to bind contaminants within soil environments. However, the differences in bacterial sources produced significant variations in mineral morphology and surface structure, which affected pollutant adsorption performance (Supplementary Table S1). Therefore, *B. velezensis*-induced biogenic vaterite is more competitive for applications such as agricultural soil improvement. Notably, the present adsorption results were obtained only from single-point static batch tests in artificial clean solution without regeneration assays, which cannot fully represent the material’s performance in complex real wastewater or soil pore water, and thus cannot be directly extrapolated to practical field remediation scenarios. The proposed model by which the two bacteria induced calcium carbonate mineralization and retained combined pollutants is shown in Figure 7.

### 4.3 Biogeochemical implications and environmental behavior of biogenic vaterite

Biogenic vaterite precipitated by microorganisms differs from chemically synthesized vaterite in phase stability. Chemogenic vaterite easily transforms into calcite or aragonite in aqueous environments, while microbially induced vaterite maintains a stable crystal structure in aquatic conditions, which is attributed to organic substances (especially proteins) embedded within crystal lattices during microbial mineralization (Liu et al., 2023; Sarkar and Mahapatra, 2010; Wang et al., 2009). The vaterite generated by *B. velezensis* possesses a specific surface area of 13.19 m^2^/g, far higher than the 2.56 m^2^/g of minerals formed by *P. putida* (Supplementary Table S1). The loose porous microstructure of *B. velezensis*-derived vaterite supplies abundant binding sites for contaminants, enabling 84.93% Cu^2+^ sequestration efficiency under a Cu^2+^ concentration of 50 mg/L (Figure 6a), which is comparable to the retention capacity of typical soil carbon components such as biochar in geochemical systems (Park et al., 2016; Tan et al., 2015).

Biogenic vaterite originates from microbial metabolic activity, which endows it good compatibility with soil matrices. Unlike artificial chemically prepared minerals, it contains no exogenous toxic additives and is mainly composed of calcium carbonate and native biological organic matter(Rodriguez-Navarro et al., 2007; Berg et al., 2014). Such properties allow biogenic vaterite to participate in natural soil mineral evolution and organic matter turnover, and it can gradually decompose under natural biogeochemical cycles with negligible risk of secondary pollution. In the Cu^2+^-TC combined pollution system, vaterite produced by *B. velezensis* maintains a Cu^2+^ sequestration efficiency above 84% (Figure 6a), whereas tetracycline retention slightly declines due to competitive occupation of binding sites. The capacity to simultaneously immobilize heavy metals and antibiotics distinguishes biogenic pedogenic carbonate from most single-component soil minerals, and partially explains the natural self-purification capacity of terrestrial soils toward composite pollutants. It is worth noting that the bacterial biomineralization behavior characterized in this study was determined under controlled laboratory conditions (LB medium, 30 °C, 180 rpm). Such standardized culture conditions were adopted to eliminate interfering factors and clearly clarify the intrinsic mineralization performance of the strain. Nevertheless, natural soil environments contain complex organic and inorganic matrices, diverse microbial communities, and variable physicochemical parameters. For this reason, the actual carbonate formation process in field soils may differ slightly from the laboratory observations, and appropriate caution is required when extending the present findings to natural soil systems. Moreover, the culture system adopted herein relies on costly LB medium, and merely 0.36 g of mineral is harvested from a 100 mL culture solution within 7 days. This indicates that indigenous soil bacteria cannot produce massive vaterite minerals within a short period under natural soil conditions. Therefore, microbially generated vaterite is not suitable for large-scale pollution remediation as artificial materials. Instead, its actual ecological function in soil environments lies in forming natural geochemical barriers *in situ* and participating in the immobilization of heavy metals and antibiotics as well as elemental cycling throughout soil profiles. Future research can adopt low-cost agricultural waste substrates to simulate natural soil nutrient conditions, clarify the formation rules of native bacteriogenic vaterite in ordinary soils, and further interpret how indigenous microbes regulate carbonate generation to constrain the migration of coexisting heavy metal-antibiotic pollutants within farmland soil ecosystems.

## 5 Conclusion

This study compared the characteristics, mineralization-related influencing factors, and pollutant sequestration behaviors of vaterite induced by *B. velezensis* and *P. putida* under identical culture conditions. As a Gram-positive strain, *B. velezensis* showed a stronger calcium carbonate mineralization capacity than the Gram-negative strain *P. putida*, with carbonate mineralization percentages of 35.06% and 27.50%, respectively. The mineralization capacity was mainly associated with EPS composition and carbonic anhydrase activity, whereas the crystal type of bacterially induced calcium carbonate was closely related to extracellular proteins. The *B. velezensis*-induced vaterite developed a loose and porous surface structure with a relatively large specific surface area. The sequestration efficiency for high-concentration Cu^2+^ and TC reached 84.37% and 80.81%, respectively. The observations obtained in this research provide basic evidence for revealing the retention mechanism of composite pollutants (copper ions and tetracycline as representatives of heavy metals and antibiotics) regulated by biogenic carbonates in terrestrial environments.

## Data Availability

The original contributions presented in the study are included in the article/Supplementary material, further inquiries can be directed to the corresponding author.
