# Supplementary material for: Mineralization mechanism and pollutant immobilization characteristics of vaterite induced by Bacillus velezensis and Pseudomonas putida
Source: Front Microbiol. 2026 Oct 13;17:1966755. doi: 10.3389/fmicb.2026.1966755 (PMC13712349; doi:10.3389/fmicb.2026.1966755)
Supplement: Supplementary file 1 [file Supplementary_file__1.docx]

Table S1 Adsorption characteristics of biogenic vaterite induced by the two bacterial strains, with BJH parameters derived from the desorption branch.

| Bacterial species | BET Surface Area | BJH Pore Volume | BJH Pore Size |
| --- | --- | --- | --- |
| *Bacillus velezensis* | 13.185 m²/g | 0.029 cm³/g | 8.428 nm |
| *Pseudomonas putida* | 2.555 m²/g | 0.008 cm³/g | 8.395 nm |

Fig. S1 Ultrastructural characteristics of *B. velezensis* and *P. putida* at day 7, showing inherent cell-wall and surface morphological differences between the Gram-positive and Gram-negative strains. (a) TEM observation of surface mineralization in *Bacillus velezensis*; (b) cellular morphology of *Bacillus velezensis*; (c) cellular morphology of *Pseudomonas putida*; (d) selected area electron diffraction (SAED) analysis of bacterial surface mineralization.

Fig. S2 X‑ray diffraction (XRD) patterns of biomimetic mineralization products. The curves correspond to the systems of ddH₂O, *Bacillus velezensis* supernatant, *Pseudomonas putida* supernatant, glucan, and bovine serum albumin, respectively. C, calcite (ICDD PDF No. 83‑0578); V, vaterite (ICDD PDF No. 74‑1867).
